# Innovative Use of RADA16 Self-Assembling Peptide (PuraBond®) as a Hemostatic Agent in Holmium Laser Enucleation of the Prostate (HoLEP): A Safety and Feasibility Study

**DOI:** 10.7759/cureus.75540

**Published:** 2024-12-11

**Authors:** Momen Sid Ahmed, Nkwam Nkwam

**Affiliations:** 1 Urology, Princess Royal University Hospital, Kings College Hospital, London, GBR; 2 Urology, King's College Hospitals NHS Foundation Trust, London, GBR

**Keywords:** bladder outlet obstruction, bleeding risks, hemostasia, holmium laser enucleation of prostate (holep), purabond®

## Abstract

Hemostasis is a critical aspect of holmium laser enucleation of the prostate (HoLEP) for benign prostatic hyperplasia (BPH). While HoLEP offers superior outcomes compared to traditional techniques, effective intraoperative and postoperative bleeding control remains a challenge. This report evaluates the feasibility and safety of PuraBond® (3-D Matrix, Ltd., Chiyoda-ku, Tokyo), a self-assembling peptide (SAP) hemostatic agent, as an adjunct for hemostasis during HoLEP.

A single case study was conducted in August 2024 on a 62-year-old male presenting with lower urinary tract symptoms (LUTSs) and a significantly enlarged prostate (152 cc). The patient underwent en-bloc low-power HoLEP under general anesthesia. Following enucleation and morcellation, initial hemostasis was achieved using bipolar diathermy, and PuraBond® was applied endoscopically to the prostatic fossa. Postoperatively, continuous bladder irrigation was initiated, and the patient was monitored for complications.

The total operative time was 120 minutes, with 66 minutes for enucleation and 14 minutes for morcellation. A total of 147.7 g of prostatic tissue was enucleated. Hemostasis was successfully achieved with the combined use of diathermy and PuraBond®, resulting in clear irrigation effluent immediately postoperatively. The patient was discharged the same day without complications. The urinary catheter was removed nine days later, and the urine remained clear throughout the recovery period. At the 28-day follow-up, the patient reported significant symptomatic improvement with no hematuria or irritative urinary symptoms. There were no adverse events or readmissions within three months post-procedure.

PuraBond® demonstrated effectiveness and safety as a hemostatic adjunct during HoLEP, providing stable hemostasis and facilitating an uneventful recovery. Its application represents a promising option for managing intraoperative and postoperative bleeding in urological procedures. Further research in larger patient cohorts is warranted to establish its efficacy and broader clinical applicability.

## Introduction

Managing intra- and postoperative bleeding in bladder outlet procedures presents a significant challenge and it can cause significant morbidity and dissatisfaction for patients. Holmium laser enucleation of the prostate (HoLEP) has become the gold standard for treating benign prostatic hyperplasia (BPH), offering superior long-term outcomes compared to transurethral resection of the prostate (TURP) [1].

Some of the conventional methods of hemostasis during HoLEP include laser coagulation, the use of intra-operative bipolar diathermy, and postoperative bladder irrigation. Despite all this, some patients are still troubled by postoperative hematuria and readmission to hospital; and in rarer cases, return to theatre for emergency bladder washout and cystodiathermy. This has prompted the exploration of hemostatic adjuncts to control bleeding more effectively. Various agents, including fibrin glues, thrombin-based products, and absorbable hemostats, have been tested with promising results [2,3].

PuraBond® (3-D Matrix, Ltd., Chiyoda-ku, Tokyo) is an innovative hemostatic agent composed of self-assembling peptides (SAPs), specifically the RADA16 sequence. Upon contact with physiological fluids, such as blood, PuraBond® forms a gel-like matrix that acts as a mechanical barrier, promoting clot stabilization without interfering with the body's natural coagulation processes. This property makes it especially suitable for delicate surgical procedures where traditional methods may cause unintended tissue damage. While PuraBond® has demonstrated effectiveness in fields like colorectal and cardiovascular surgeries [4],its use in urological procedures, such as HoLEP, remains underexplored. This case study aims to assess the safety and feasibility of PuraBond® for hemostasis during HoLEP, potentially offering a useful alternative to traditional methods.

## Case presentation

**Figure 1:**
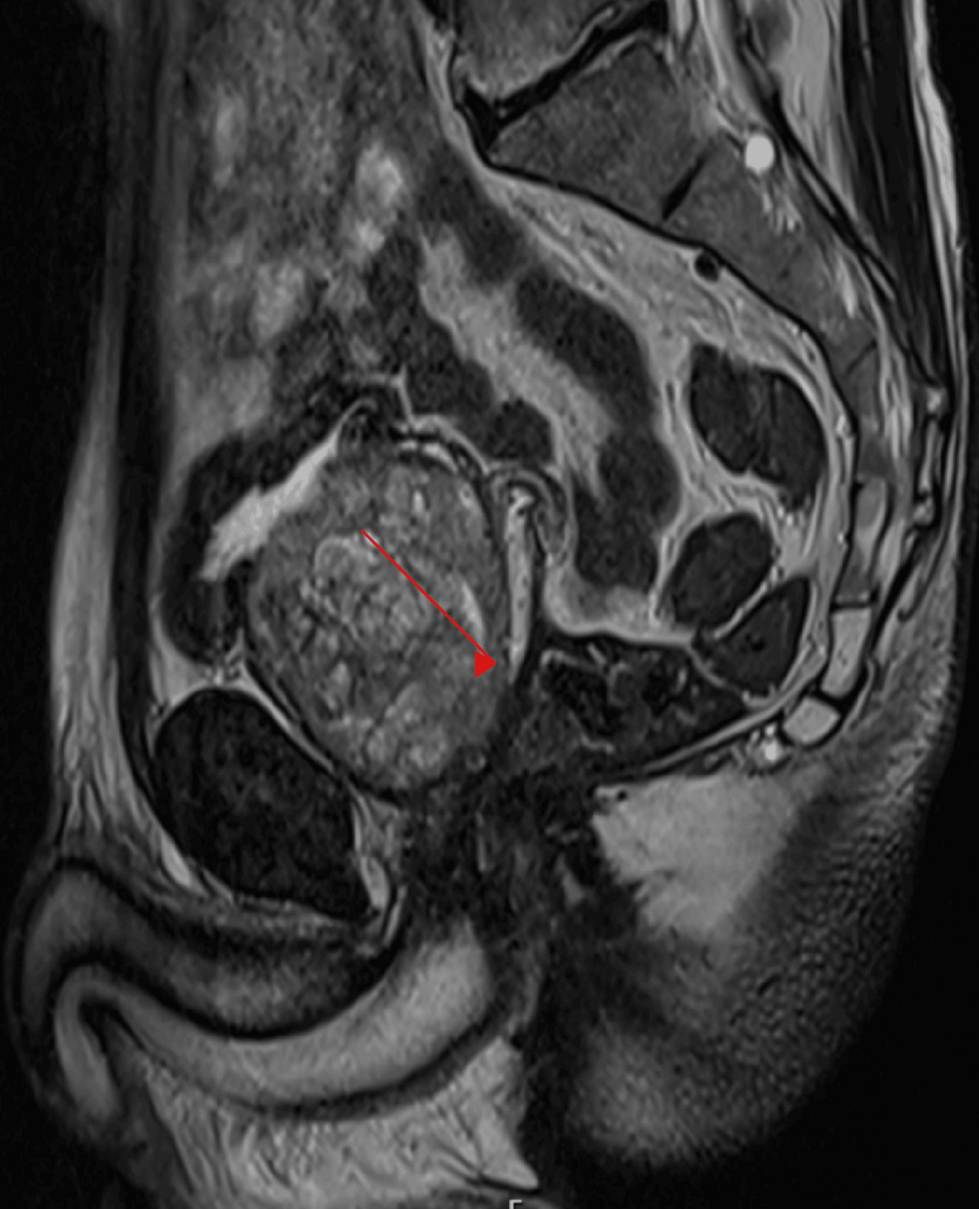
Pre-operative MRI sagittal view showing enlarged prostate (red arrow)

**Figure 2:**
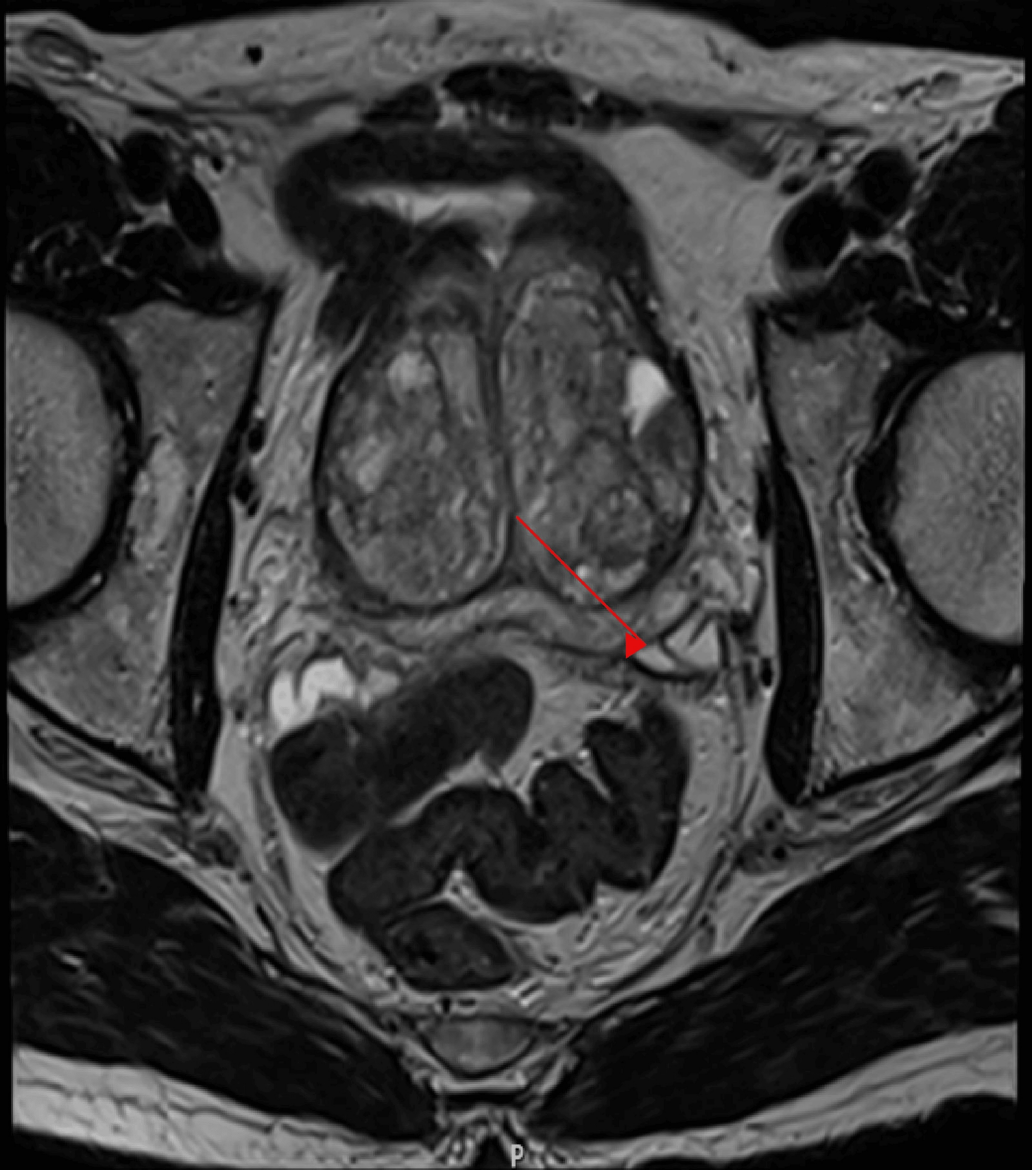
Pre-operative MRI axial view showing enlarged prostate (red arrow)

This single case study was conducted in August 2024 at a large district general hospital. The patient was a 62-year-old male with a history of lower urinary tract symptoms (LUTSs) and prostate cancer under active surveillance for Gleason 3+3 = 6 T2N0Mx, low-risk classification. The patient developed BPH progression with a pre-operative International Prostate Symptom Score (IPSS) of 17 out of 35 and a quality-of-life score of 3 out of 6. The maximum urinary flow rate (Q-max) was 2.5 mL/s with a post-void residual (PVR) volume of 200 mL. Imaging revealed a significantly enlarged prostate, with a volume of 152 cc, and a prostate-specific antigen (PSA) level of 8.32 ng/mL as of March 2023 (Figures 1, 2).

The patient had no significant comorbidities (ASA 1) and underwent en-bloc low-power HoLEP using a 600μm laser fiber (18Hz/2,200 mJ) and 39.6W of power under general anesthesia with muscle paralysis, which is the standard of care in our unit. Following the removal of the prostatic tissue, hemostasis was initially achieved using a bipolar button electrode to allow clear enough views for the application of PuraBond®. The bladder was then emptied via the laser scope and an empty Ellik evacuator (UROMED, Oststeinbek, Germany) was used to expel the remaining irrigation fluid from the bladder and also to fill the bladder partly with air. This will also prompt any fluid residing in the empty prostate fossa to be pushed back into the bladder, leaving a dry field as much as possible for the application of PuraBond®.

A 22 French rigid cystoscope was then inserted with the tip just beyond the veru montanum in the prostatic fossa and 5 mL of PuraBond® was instilled under direct vision via a dedicated 6Fr applicator. The procedure concluded with the insertion of a 22Fr/3-way catheter, and very slow continuous bladder irrigation was initiated. The urine color on very slow irrigation immediately postop was very lightly bloodstained (Video 1).

**Video 1:** PuraBond® application A 5 mL of PuraBond® administered under direct visualization using a dedicated 6Fr applicator

**Figure 3:**
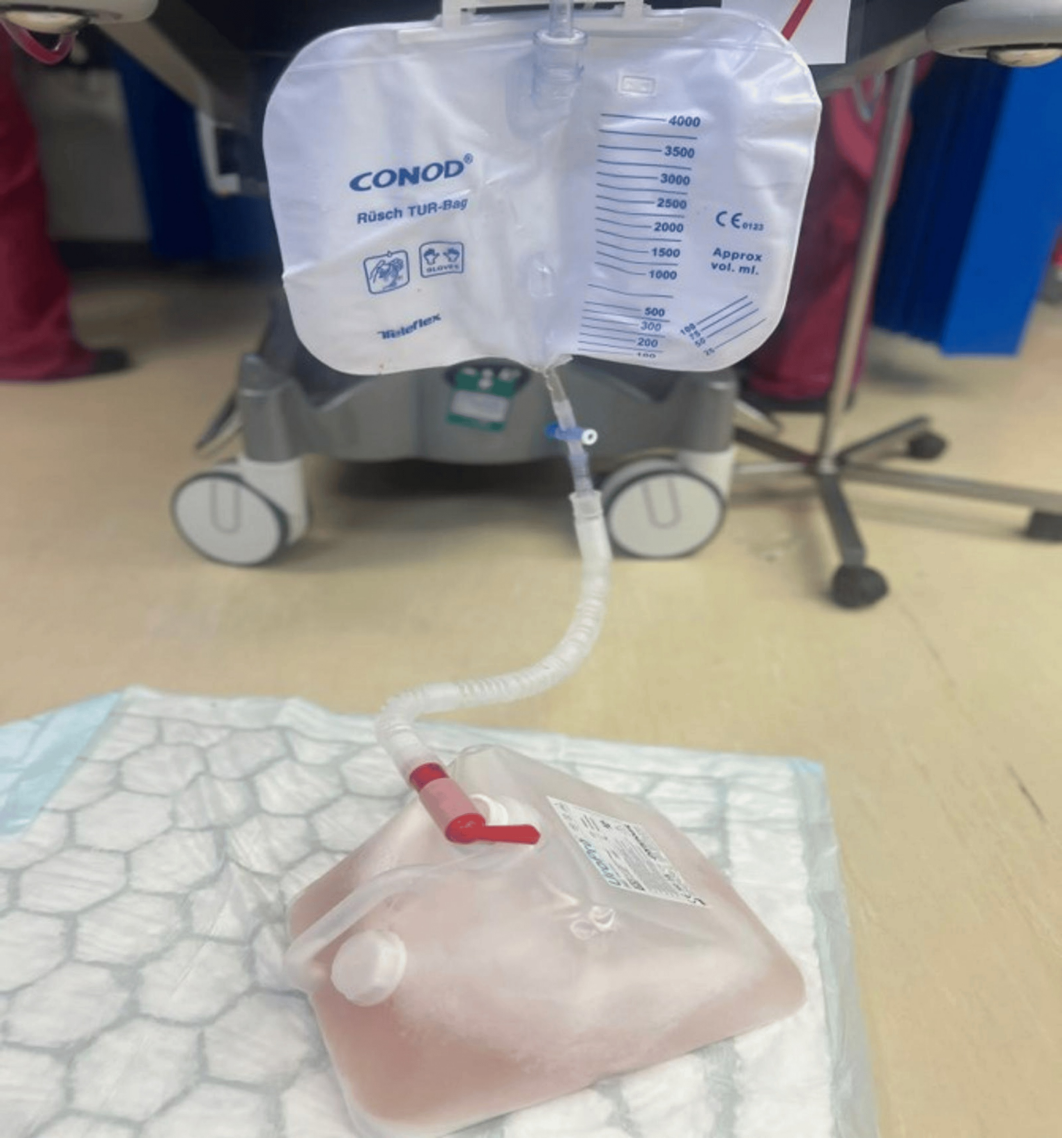
Urinary catheter bag Urine color minimally blood stain on very slow irrigation postoperatively

The patient's continuous irrigation showed clear urine throughout the postoperative period (Figure 3).

In this case, the patient successfully underwent HoLEP with the application of PuraBond® for hemostasis. The procedure was notable for a total operative time of 120 minutes, which included 66 minutes for enucleation and 14 minutes for morcellation. The patient's prostate was measured at 152cc pre-operatively and yielded 147.7 g of enucleated benign tissue. Hemostasis was achieved effectively, both through bipolar diathermy and the subsequent application of PuraBond®. The patient had an uneventful postoperative course, with continuous bladder irrigation showing clear urine immediately after surgery. The patient was discharged on the same day without any postoperative complications.

At follow-up one week later, the catheter was successfully removed, and the patient continued to report clear urine. At the 28-day follow-up, there were no reports of complications, with the patient’s LUTSs significantly improved, and no signs of hematuria were observed. There has been no readmission to the hospital within three months of the procedure, and the patient did not report any adverse side effects.

## Discussion

Controlling hemostasis during HoLEP is essential to allow clear views for morcellation and to avoid complications such as hematuria and clot retention. Traditional methods, such as diathermy and bladder irrigation, may be insufficient, particularly in patients with large prostates or those on anticoagulants. In this case, PuraBond® provided an effective alternative for rapid hemostasis, with no postoperative complications observed. The application of PuraBond® was quick and resulted in excellent hemostasis. The patient’s recovery was uneventful, and he was discharged the same day with clear urine. The catheter was removed successfully after one week, and no adverse events related to PuraBond® were reported during follow-up.

The use of PuraBond® in this case aligns with its effectiveness in other surgical fields. Its mechanical action - forming a stable barrier over bleeding sites while preserving tissue integrity - makes it a valuable adjunct in open and laparoscopic-assisted urological surgeries [5]. However, from this anecdotal experience, we cannot derive any conclusion about its efficacy but at short to medium-term follow-up we have demonstrated that PuraBond® is feasible to apply endoscopically and safe to use directly in the prostatic fossa. As this study reports a single case, further research involving larger patient cohorts and longer follow-up as well as possible comparative analyses with traditional hemostatic methods is needed. The outcome of this case suggests that PuraBond® is a promising hemostatic agent during HoLEP and may be beneficial in other urological procedures as well.

## Conclusions

We report the first-ever use of PuraBond® as a hemostatic agent during low-power HoLEP surgery. This case highlights the potential utility of PuraBond® as a hemostatic adjunct during HoLEP, demonstrating its feasibility, safety, and effectiveness in achieving rapid hemostasis. The mechanical action of the SAPs in forming a stable gel matrix provided superior control of bleeding without interfering with the natural coagulation process. Additionally, the quick application and ease of use make it a promising tool in the management of challenging hemostasis scenarios, particularly in patients with large prostates or those at higher risk of bleeding complications. While this single case yielded excellent short- and medium-term outcomes, including an uneventful recovery and significant symptom improvement, further studies involving larger cohorts and longer follow-ups are required to validate these findings. The incorporation of PuraBond® into routine practice could potentially enhance patient outcomes and reduce complication rates in bladder outlet procedures, paving the way for its broader applicability in urological surgeries.
